# A Review of *Quercus infectoria* (Olivier) Galls as a Resource for Anti-parasitic Agents: In Vitro and In Vivo Studies

**DOI:** 10.21315/mjms2019.26.6.3

**Published:** 2019-12-30

**Authors:** Nik Nor Imam Nik Mat Zin, Wan Nur Addiena Wan Mohd Rahimi, Nurhidanatasha Abu Bakar

**Affiliations:** School of Health Sciences, Universiti Sains Malaysia, Kubang Kerian, Kelantan, Malaysia

**Keywords:** Quercus infectoria, anti-parasitic activity, phytochemicals, toxicity activity, anti-oxidant activity

## Abstract

Parasitic diseases represent one of the causes for significant global economic, environmental and public health impacts. The efficacy of currently available anti-parasitic drugs has been threatened by the emergence of single drug- or multidrug-resistant parasite populations, vector threats and high cost of drug development. Therefore, the discovery of more potent anti-parasitic drugs coming from medicinal plants such as *Quercus infectoria* is seen as a major approach to tackle the problem. A systematic review was conducted to assess the efficacy of *Q. infectoria* in treating parasitic diseases both in vitro and in vivo due to the lack of such reviews on the anti-parasitic activities of this plant. This review consisted of intensive searches from three databases including PubMed, Science Direct and Scopus. Articles were selected throughout the years, limited to English language and fully documented. A total of 454 potential articles were identified, but only four articles were accepted to be evaluated based on inclusion and exclusion criteria. Although there were insufficient pieces of evidence to account for the efficacy of *Q. infectoria* against the parasites, this plant appears to have anti-leishmanial, anti-blastocystis and anti-amoebic activities. More studies in vitro and in vivo are warranted to further validate the anti-parasitic efficacy of *Q. infectoria*.

## Introduction

Parasites exist in two different forms, the unicellular protozoa and the multicellular metazoa that acquire nourishment and other basic needs from their hosts through parasitism. Most parasites reproduce asexually and sexually in a single host species (monoxenous life cycle), while the other parasites reproduce in multiple host species (heteroxenous life cycle) assisted by a living carrier called vector (1). Three major classes of parasites known to cause diseases in humans are protozoa (kingdom Protista), helminths and arthropods (kingdom Metazoa) (2).

Malaria, the most common parasitic disease caused by *Plasmodium* species affects many lives and caused 435,000 deaths worldwide in 2017 (3). Lymphatic filariasis, soil-transmitted helminthiasis, schistosomiasis, trachoma and/or onchocerciasis, which are known as neglected tropical diseases (NTDs) caused 1.6 billion deaths in 2015 (4). Many parasitic zoonoses such as cryptosporidiosis, toxoplasmosis and leishmaniasis also result in varying morbidity and mortality among vulnerable populations as well as people suffering from clinical symptoms (i.e. various forms of immunosuppression rather than immunocompetence) (5–7). *Entamoeba histolytica*, a major pathogen from a group of Amebae (protozoa) induces amoebic dysentery and liver abscess that mainly affects areas with poor sanitation, typically through faecal-contaminated water or food (8).

Parasites have unique characteristics whereby parasite-host interactions can be easily accessible due to the similarities in most molecular and biochemical properties (9, 10). Hence, it is not surprising that parasites could easily adapt in human hosts for many years and thus, being responsible for parasitic diseases (11). The evolution of parasite mutation through antigenic profile changes and immune evasion mechanisms also increases parasite survival from the host’s defence mechanism, leading to life-threatening diseases (12). Chemotherapy remains central to both clinical treatment and disease control whereby the efficacy has constantly been threatened by the emergence of a single drug- or multidrug-resistant parasite populations (12). Other strategies include the development of newly modified former drugs with novel mechanisms to avoid cross-resistance with existing less potent drugs have also been hindered by the challenges of environmental implications as well as vector threats (13). The high expenditure on the development of novel anti-parasitic drugs is another challenge preventing future treatment and control of many parasitic diseases to flourish (14).

The discovery of natural products derived from living organisms such as microorganisms and plants has become one of the major approaches to tackle the parasitic problem (15, 16). The discovery of moxidectin and artemisinin as effective modern medicines is a remarkable example of the therapeutic value derived from natural products first used traditionally (16). Moxidectin and avermectins isolated from a soil bacterium *Streptomyces avermitilis* have been approved to treat river blindness effectively, which is one of the NTDs transmitted by the bite of blackflies (2). Previously, the active ingredient quinine from the bark of the Cinchona tree was used for the treatment of malaria (17). Currently, artemisinin has been established as a highly potent anti-malarial drug, which is co-formulated with other partner drugs to be used as the first-line of treatment for *P. falciparum* malaria (18). Other bioactive constituents from higher plants such as alkaloids, terpenes and phenolics have also been tested extensively against various parasites and have shown to be potential new drug leads and may have positive impacts on future drug developments (19).

*Q. infectoria* Olivier (family Fagaceae), a plant of about two metres high, is widely distributed across the Mediterranean area (Greece, Asia Minor, Syria and Iran) (20). Galls from this plant are a special natural product resulting from a parasitic interaction between the plant and the insect known as a gallfly or *Cynips gallae-tinctoriae* (20). The galls, known as ‘manjakani’ in Malaysia and ‘majuphal’ or ‘machakai’ in India, have been used for ages as a traditional medicine to treat various ailments. Until today, the *Q. infectoria* galls have been utilised by Malay women for post-partum medication and as health supplements (*jamu*). Some also claimed that the oral administration of jamu from the galls helps to improve blood flow, speed up the contraction of the uterus and tighten the vagina, as well as encourage bowel movement (21). Besides, the galls have also been used in Thailand traditional medicine for treating stomach ache (22, 23) while in India, the galls extract has been used for oral care as mouth wash, dental powders and for treatment of toothache (24, 25). The galls are rich with tannins, the phenolic compounds, which are thought to have an astringent effect and have also been used in topical therapies for skin lesions and inflammation in Chinese medicinal herb (24).

The medicinal values of *Q. infectoria* with anti-diabetic (26), anti-tremorine (27), anti-inflammatory (28) and astringent activities (25), as well as having a broad spectrum of anti-microbial properties such as anti-bacterial (29–31), anti-viral (32) and anti-fungal activities (33, 34) have been highlighted as the outstanding potentials of the galls. The diverse anti-microorganism activities of *Q. infectoria* galls have encouraged researchers to further study the biological activities of the plant. This present work is a review of information on the anti-parasitic activities of *Q. infectoria* galls in vitro and in vivo that could be used by researchers to comprehensively investigate the molecular mechanisms underlying the anti-parasitic effects of the galls.

## Methods

### Search Strategy

Three electronic databases used as sources for literature review were PubMed, Science Direct and Scopus from 1990 until 7 January 2019. As the purpose of this review was to systematically evaluate the efficacy of *Q. infectoria* galls in treating parasitic diseases, research articles conducted in vitro, in vivo or both were targeted.

The keywords used as the search terms were as follows:

- *Quercus infectoria* and anti-microbial
- *Quercus infectoria* and anti-parasitic
- *Quercus infectoria* and anti-protozoal
- *Quercus infectoria* and anti-leishmanial
- *Quercus infectoria* and anti-helminthic
- *Quercus infectoria* and anti-malarial
- *Quercus infectoria* and anti-amoebic
- *Quercus infectoria* and anti-blastocystis
- *Quercus infectoria* and anti-plasmodial
- *Quercus infectoria* and anti-trypanosomal
- *Quercus infectoria* and anti-giardial
- *Quercus infectoria* and anti-schistosomial
- *Quercus infectoria* and anti-acanthamoeba

### Research Article Selection and Evaluation

Search results were limited to fully documented articles in English without restriction on the date of publication.

Inclusion criteria:

- Full-text articles
- In vitro studies related to parasites
- In vivo studies related to parasites
- Intervention subject, with only *Quercus infectoria*

Exclusion criteria:

- Irrelevant titles and abstracts
- Duplicated studies
- Reviews
- Subject index
- News
- Case studies
- Poor methodology
- Not related to any parasites

Two independent reviewers screened the articles based on the inclusion and exclusion criteria stated above. For the first screening, the related articles were screened based on their titles and abstracts. Next, the remaining papers were checked for duplications and those with exclusion criteria were also eliminated. Finally, the selected full-text articles were checked by another reviewer according to the inclusion criteria for final validation. The extracted data are summarised as shown in Table 1, Table 2, Table 3 and Table 4.

## Results and Discussion

### Summary of Included Studies

**Figure 1:**
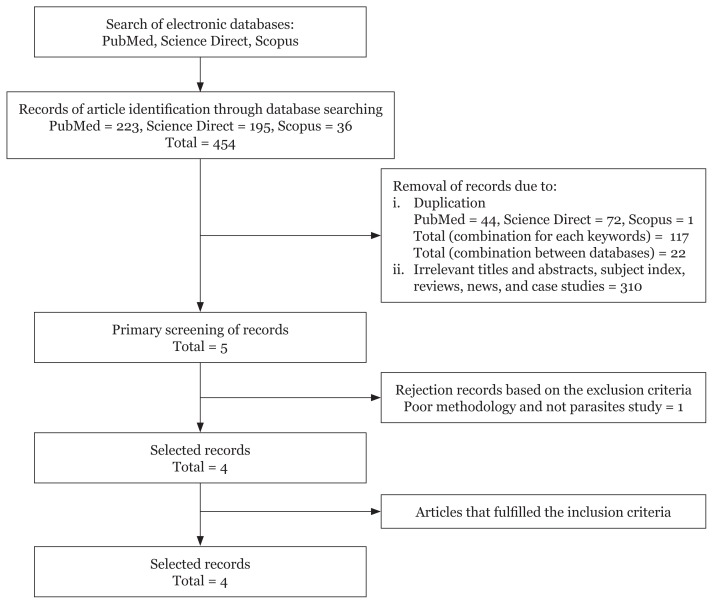
The selection process of the articles used in this systematic review

A total of 454 potential articles were obtained using keyword search from PubMed, Science Direct and Scopus databases. A total of 139 duplicates were removed. From the remaining 315 articles, five articles were selected after those potentially not related to the criteria including irrelevant titles and abstracts, reviews, subject index, news and case studies were removed. Finally, four full-text articles, which fulfilled the inclusion and exclusion criteria were accepted after further evaluation of the five articles. Figure 1 illustrates the article selection based on the inclusion and exclusion criteria.

Of the four selected articles, five anti-parasitic studies conducted consist of three in vitro and two in vivo studies and appeared in the literature between 2004 and 2016. Two of the four articles were conducted in Southeast Asia (Thailand), while the remaining two articles were conducted in Middle East countries (Turkey and Iran) (Table 1). All of the articles reported the use of methanol as a solvent for plant extraction, and two articles reported the use of other solvents such as n-hexane (23, 35) and dichloromethane (23). Nevertheless, the in vitro anti-oxidant and toxicity studies presented in the selected articles were also included in this review to support the efficacies and the anti-parasitic activities of *Q. infectoria*.

A total of three in vitro anti-parasitic studies were conducted by Sawangjaroen and Sawangjaroen (23), Ozbilgin et al. (35) and Kheirandish et al. (36), while two in vivo studies were carried out by Sawangjaroen et al. (37) and Kheirandish et al. (36) (Table 4). Three types of parasites were subjected to the studies: *Entamoeba histolytica* (37), *Blastocystis hominis* and *Blastocystis* spp (23, 35) and *Leishmania major* (36). For the in vivo studies, mice models were exclusively used, specifically female Swiss albino mice and male BALB/c mice.

### Preliminary Phytochemical Analysis of the Crude Extracts of the Q. infectoria Galls

The activities of the plant could be influenced by various factors such as the plant species and the parts of the plant, sex of cultivars, geographical origin, harvesting time as well as climatic conditions which can also interrupt the reproducibility of the results (38). As the demand for herbal medicines increases due to minimal adverse effects seen in humans compared to synthetic drugs (39), it is necessary to characterise herbal medicines to ensure their quality, efficacy and safety. To verify and ensure that the correct plant was assessed throughout this review, it is crucial for the plant in the selected articles to be authenticated. This is because the sources of medicinal plants may vary according to the respective countries (39). In this review, all four studies reported the authentication of *Q. infectoria* galls with the voucher specimen that was made in their respective countries (Thailand, Turkey and Iran) (23, 35–37) (Table 1).

From the four selected articles, a total of three solvents (methanol, n-hexane and dichloromethane) were used and the percentage yield of the extracts of *Q. infectoria* galls was identified as indicated in Table 1. Briefly, a conventional maceration method was carried out where *Q. infectoria* galls were extracted at an appropriate ratio (1:3) of gall powder to solvent, filtered and evaporated to dryness to form powdery extracts. The methanol extract was tested in all of the selected articles. This extract represented the highest yield at 50.1%, 46.7% and 45.0% in three out of four articles (23, 36, 37). The percentage yield recorded for the methanol extract has no significant difference between all studies, indicating that the extraction method does not influence the total crudes obtained (*P* > 0.05) (40).

Phytochemical composition of plant sources is the primary identification for the fundamental understanding of the acting mechanism of the crude extracts (41). Unlike primary metabolites, which are required for maintaining growth as well as proliferation, secondary metabolites (also known as bioactive metabolites) not only function as a protectant under damage due to stress and harsh environment, but also serve as a weapon against several microorganisms such as bacteria, fungi, parasite and higher organisms (42). A study by Kheirandish et al. (36) was conducted to determine the bioactive compounds in the *Q. infectoria* methanol extract before the anti-leishmanial test. Determination of the total percentage of phenolic and flavonoid compounds was carried out using Folin-Ciocalteau and Dowd methods, respectively. Quercetin and gallic acid were also analysed using high performance liquid chromatography (HPLC). The results showed that both phenolic and flavonoid compounds exist in the methanol extract, however, in comparison, the presence of the phenolic compound (57.50%) is higher than the flavonoid (1.86%) in the methanol extract (Table 2). The values of quercetin and gallic acid were 0.0064% and 0.22%, respectively (Table 2). A similar study conducted by Baharuddin et al. (33) also showed that the presence of phenolic compound in both methanol and aqueous extracts of *Q. infectoria* galls using GC-mass spectrophotometry (GC-MS) analysis, thus proves that the main compounds in the methanol extract of *Q. infectoria* galls were the phenolic compound (expressed as 0.22% of gallic acid) followed by flavonoid (expressed as 0.0064% quercetin).

The main bioactive constituent found in both methanol and aqueous extracts of *Q. infectoria* galls was pyrogallol or hydrolysable tannin from phenolic compound (33). Pyrogallol is known to be associated with many biological activities and implicated in many anti-microbial activities such as anti-bacterial, anti-candidicidal and anti-fungicidal (43). Interestingly, Abdullah et al. (44) recently revealed that different semi purifed fractions of the aqueous extract of *Q. infectoria* have similar polyphenolic compositions such as gallic acid and digallate as well as ellagic acid, syringic acid and theogallin, which have also been detected using liquid chromatography mass spectrometry (LC-MS), implying the richness of phenolic compounds in the galls. Similarly, ethanol extract of *Q. infectoria* galls has also been shown for their high content of phenolic compounds such as p-Hydroxybenzoic, pyrogallol, catechol, caffeine and gallic acid (31). Flavonoid compound found in the ethanol extract with the main flavonoid compound was naringin, rutin, rosmarinic, quercetrin and quercetin (31). Taken together, the above studies suggest that the anti-parasitic activities of the *Q. infectoria* gall extracts could be mediated through the presence of rich contents of phenolic and flavonoid compounds.

### Screening of Toxicity and Anti-Oxidant Activities of Q. infectoria

Scientific evidence to establish the safety of herbal medicines for human consumption is important to assure consumers of unwanted side effects (45). There are two methods used for toxicity testing of the extracts of *Q. infectoria* galls found in the chosen articles (Table 3): brine shrimp lethality test (BSLT) and cytotoxicity test. The BSLT test carried out by Ozbilgin and his colleagues (35) showed that the LC_50_ value (a lethal concentration that kills 50% of the shrimp population) of the methanol extract was considered as toxic according to Meyer’s toxicity index (46). However, the n-hexane extract was reported as non-toxic even though the LC_50_ value of the extract was not indicated in the study.

In the study by Kheirandish et al. (36), the methanol extract of the galls was assessed for both cytotoxicity and anti-oxidant activities. The extract was considered to possess a promising anti-oxidant activity with an IC_50_ value (a concentration that inhibits oxidation by 50%) of 30.78 μg/mL while butyl hydroxy toluene (BHT) (a standard anti-oxidative agent) gave an IC_50_ value of 31.50 μg/mL (Table 3). The finding was supported by the high percentage of phenolic and flavonoid compounds in the methanol extract, which enhanced anti-oxidant activity by trapping, scavenging and eliminating free radicals and by chelating metals (47, 48). The cytotoxicity activity of the extract on macrophage cells was also evaluated using 3-(4,5-dimethylthiazol-2-yl)-2,5-diphenyltetrazolium bromide (MTT) assay (36). The CC_50_ value (a concentration that reduces cell viability by 50%) shows no cytotoxic effect of the extract on the normal macrophage cells.

The contradictory results obtained from the BSLT and cytotoxicity tests of the methanol extract might be due to the differences of the in vitro models (i.e. shrimps and normal human macrophage cells), concentrations of the plant extract, and geographical origin of the plant sources that were used in the two studies (45).

### Anti-Parasitic Activities of the Crude Extracts of the Quercus infectoria Galls Against Intestinal and Intracellular Protozoa

A total of four articles consisting of three in vitro and two in vivo studies conducted between 2004 and 2016 were examined for the effects of the crude extracts of *Q. infectoria* galls against the parasites. The three in vitro studies were conducted by Sawangjaroen and Sawangjaroen (23), Ozbilgin et al. (35) and Kheirandish et al. (36), while two in vivo studies were carried out by Sawangjaroen et al. (37) and Kheirandish et al. (36).

The study of the *Q. infectoria* methanol extract on caecal amoebiasis-infected mice was conducted by Sawangjaroen et al. (37) using *Entamoeba histolytica*, an intestinal pathogenic protozoan isolated from the infected patient at Maharaj Hospital, Nakorn Srithamarat, Thailand (Table 4). This experiment was the first to use a mice model in contrast to previous studies, which generally utilised rat models in anti-amoebic tests (37, 49). They reported that female Swiss albino mice fed with 500 mg/kg of the extract showed a higher number of mice cured from amoebiasis (26%) compared to the untreated control group after five consecutive days of treatment. The severity of the mice caecal content and the caecal wall lesions were also reduced in comparison to the untreated mice. Metronidazole was used as a standard control in the experiment (showed 100% curing of *E. histolytica*-infected mice at 125 mg/kg) to compare the performance of the extract against the parasite. Although there was a significant difference in the anti-amoebic activity between the extract and the standard drug (*P* < 0.05), the extract could be said to at least help in reducing severity occurred in the mice intestine. Thus, the researchers concluded that the *Q. infectoria* methanol extract appeared to have an anti-amoebic potential against caecal amoebiasis in mice.

Blastocystis spp. (e.g. B. hominis) is a common protozoan detected in the human intestine and primarily recognised as a normal intestinal flora but can result in diarrhoea, abdominal pain and vomiting in immunosuppressed hosts (50, 51). Claimed as an alternative for diarrhoea treatment in high-risk countries (52), two in vitro studies were conducted by Sawangjaroen and Sawangjaroen (23) and Ozbilgin et al. (35) using the *Q. infectoria* extracts against different virulent strains of Blastocystis spp. isolates (Table 4). They reported that the inhibitory effects of the extracts were dose-dependent. At 2,000 μg/mL, the methanol extract of the *Q. infectoria* galls evaluated by Sawangjaroen and Sawangjaroen (23) killed 67% (KC_50_ = 1248 μg/mL) and inhibited 76% (IC_50_ = 171 μg/mL) of B. hominis. Later, Ozbilgin et al. (35) investigated which type of extract (n-hexane and methanol) showed the best performance against the parasite. They showed that the methanol extract was the most effective to inhibit the in vitro growth of Blastocystis spp. isolates (EC_50_ = ~336.8 μg/mL) compared to the n-hexane extract. From the two studies using the methanol extract, the study done by Sawangjaroen and Sawangjaroen (23) showed the most active methanol extract in comparison to the methanol extract prepared by Ozbilgin et al. (33).

Leishmaniasis is one of the seven most important tropical diseases caused by an obligate intracellular parasite from the genus of *Leishmania* and transmitted to humans by the bite of the arthropod infected female sand fly, mainly *Phlebotomus* in the Old World and *Lutzomyia* in South America (53, 54). The vector-borne disease is manifested by three clinical forms: visceral, cutaneous and mucocutaneous leishmaniasis (55), whereby cutaneous leishmaniasis (CL) is associated with ulcers, cauliflower-like masses or nodules (56). Therefore, both in vitro and in vivo anti-leishmanial studies were evaluated by Kheirandish et al. (36) against promastigote (the virulent form with flagella) and amastigote forms (non-flagellated) of *L. major*. The in vitro study showed that the amastigote form of *L. major* was more sensitive to the *Q. infectoria* methanol extract compared to the promastigote form. They also reported that the methanol extract demonstrates promising results compared to a reference anti-leishmanial drug, glucantime meglumine antimoniate (MA) in an in vitro study (Table 4). Similar results were obtained in the in vivo study, whereby the number of parasites and parasite load significantly decreased (*P* < 0.05) after treatment with 10 mg/kg and 20 mg/kg of the methanol extract compared to the untreated control group with no decrease in the number of parasites. Moreover, the diameter of the tail lesion affected by *L. major* also reduced to about 0.86 cm, 4.20 cm and 5.11 cm from 14.14 cm (the untreated control size of the lesion) at the concentration of 20 mg/kg, 10 mg/kg and 5 mg/kg of the extract after four weeks of treatment, respectively. Healing rate of the lesion in infected male BALB/c mice was observed after four weeks of treatment with 20 mg/kg extract (91.6% recovery) compared to MA (66.6%) indicating that the extract was highly potent in reducing leishmaniasis. They presumed that the high amount of phenolic compound (57.50%) might be responsible for the anti-leishmanial activity of the extract. Consistent with the previous study, inhibition of *L. tropica* and *L. major* was correlated with the content of the phenolic compound of Tunisian olive tree (57).

Although the mechanism of anti-parasitic action of *Q. infectoria* has not yet been elucidated, its ability to curb protozoa-causing diseases is thought due to the vast variety of bioactive constituents, which are responsible for the disruption of amino acid production required for parasitic growth during translation pathway (36). The bioactive compounds present have also exhibited an anti-microbial effect by damaging the cell membrane, which also seems to be the mode of anti-parasitic action of *Q. infectoria* (36).

All in vitro studies from the selected articles showed that *Q. infectoria* has the potential in decreasing the parasite growth evaluated by the IC_50_ and EC_50_ values and in killing the parasites based on the KC_50_ value against blastocystis and leishmaniasis. Furthermore, the in vivo studies also showed similar results in which *Q. infectoria* was able to inhibit parasites causing amoebiasis and leishmaniasis. The findings provide a fundamental view that *Q. infectoria* could be further investigated for searching potential bioactive metabolites responsible for anti-parasitic activities such as anti-malarial activity.

## Strength and Limitations

This systematic review is among the first to describe in detail the in vitro and in vivo studies on the efficacy of *Q. infectoria* galls against the parasites. It prioritises the anti-parasitic aspects using the *Q. infectoria* crude extracts whilst various other studies concentrated more on other microorganisms and diseases. Vaccines still do not work for many parasites, causing most parasitic diseases to be neglected. This review has a huge advantage in providing the knowledge gap on the *Q. infectoria* capability, which can be used as a reference for new research to support the efficacy of *Q. infectoria* by elucidating the anti-parasitic activities against several other parasites. This review also identified several limitations. Several studies did not clearly state the proper methodology of the plant extraction (e.g. ratio of gall powder to solvent) and the final percentage of yield obtained, hence restricted future studies to prepare and produce appropriate stocks of the crude extracts. Furthermore, the phytochemical constituents of several extracts had not been revealed qualitatively and quantitatively because different solvents might influence the outcome of the constituents, which might be responsible for the anti-parasitic activity. Moreover, only a few studies identified the toxicity of the plant. It is important to evaluate in detail the safety of *Q. infectoria* galls both in vitro and in vivo before different tests could be carried out and before the plant could be implemented as an alternative, promising and safe anti-parasitic agent. Lastly, some experiments against virulent parasites were only performed in vitro and not in vivo and vice versa, restricting the promising results against the anti-parasitic activity of the plant.

## Recommendation

Based on this review, we suggest more rigorous studies using other different parasites both in vitro and in vivo to support the efficacy of *Q. infectoria* galls. This plant might have activities and effects against many other parasites without compromising the plant status, extraction method, phytochemistry as well as the toxicological aspects to maintain the reproducibility and accuracy of the overall studies.

## Conclusion

There is still insufficient evidence to draw a definitive conclusion on the efficacy of *Q. infectoria* galls against the parasites tested; however, it appears to possess the anti-leishmanial potential against *L. major* shown in both in vitro and in vivo studies. The plant is also a novel candidate to treat other tropical parasitic diseases such as blastocystis and amoebiasis. Therefore, the available data from the in vitro and in vivo tests might warrant further studies to provide more details and clearer overview of the anti-parasitic properties of the *Q. infectoria* galls.

## Figures and Tables

**Table 1:** The information on *Q. infectoria* sources, authentication and processing of *Q. infectoria* gall extracts

Article	Plant source	Authentication	Solvent	Methodology	Percentage yield	Comment
Sawangjaroen et al. (37)	Medicinal plant store in Thailand	The Prince of Songkla, University Herbarium, Department of Biology, Faculty of Science, Prince of Songkla University, Thailand (Voucher no.: K. SAWANGJAROEN 2 (PSU))	Methanol	The crude extract was prepared using a maceration method Galls were extracted with the solvent at a 1: 3 ratio The extract was filtered and evaporated to dryness with a rotary evaporator (55 °C) The extract was stored at 4 °C until being use	Methanol: 46.7	The extract was further tested for the in vivo anti-parasitic activity.
Sawangjaroen and Sawangjaroen (23)	Medicinal plant store in Thailand	The Prince of Songkla, University Herbarium, Department of Biology, Faculty of Science, Prince of Songkla University, Thailand. (Voucher no.: K. SAWANGJAROEN 2 (PSU))	Dichloromethane Methanol n-hexane	The crude extracts were prepared using a maceration method A ratio of galls powder to respective solvent was 1:3 The extracts were filtered and evaporated to dryness with a rotary evaporator at 55 °C The extracts were dissolved in dimethylsulfoxide (DMSO) and stored at 4 °C until being use	Dichloromethane: 0.2 Methanol: 50.1 n-hexane: 0.2	The antiparasitic activity of the n-hexane and dichloromethane extracts was not determined due to insufficient supply of the extracts (< 0.5%)
Ozbilgin et al. (35)	Yagcilar village, Manisa province in western Turkey	Herbarium of Celal Bayar University, School of Science and Letters, Department of Biology. (Voucher no.: Not stated)	Hexane Methanol	The crude extracts were prepared and extracted under stirring technique Organic phases were filtered (0.45 μm) and distilled in vacuo	ND	All extracts were tested for the in vitro cytotoxicity and antiparasitic activities
Kheirandish et al. (36)	Rural regions of Khorramabad district, Lorestan Province, west of Iran, in September 2013	Razi Herbal Medicine Research Center, Lorestan University of Medical Sciences, (Khorramabad, Iran). (Voucher no.: RH 1165)	Methanol	The crude extract was prepared using a maceration method Galls were extracted with the solvent and soxhlet extractor at 50 °C The extract was evaporated using a rotary evaporator The extract was stored at 4 °C for later use	Methanol: 45.0	The extract was tested for the in vitro antioxidant and cytotoxicity activities as well as for the in vitro and in vivo studies of the antileishmanial activity

ND = Not determined

**Table 2:** The secondary metabolites in the methanol extract of *Q. infectoria* galls

Article	Chemical constituents	Percentage of yield
Kheirandish et al. (36)	Phenolic	57.5000
	Flavonoid	1.8600
	Quercetin	0.0064
	Gallic acid	0.2200

**Table 3:** The summary of the anti-oxidant and toxicity effects of different *Q. infectoria* solvents on the brine shrimps test and cytotoxicity assay

Article	Extract	Antioxidant activity	Toxicity effects		Interpretation
	
		IC_50_ (μg/mL)	BSLT test LC_50_ (μg/mL)	MTT assay CC_50_ (μg/mL)	
Ozbilgin et al. (35)	Methanol	ND	190.86	ND	The extract was considered as toxic (LC_50_ < 1000 μg/mL is considered as toxic based on Meyer’s toxicity index)
	n-hexane	ND	NR (non-toxic)	ND	No value for LC_50_ for the extract was shown. However, no toxicity was reported for the extract in the article
Kheirandish et al. (36)	Methanol	30.78	ND	210.75 5.18	The extract showed a considerable antioxidative activity and not significant when compared with the positive control [butyl hydroxy tuloene (BHT) standard] which has an IC_50_ value of 31.50 μg/mL (*P* > 0.05). The extract had no cytotoxic effect on the normal human macrophage cells

Notes: ND = not determined, NR = not reported

Half maximal inhibitory concentration (IC_50_) is the concentration required to inhibit 50% oxidation (free radical). Half median lethality concentration (LC_50_) is the concentration required for killing 50% shrimps. Half maximal cytotoxicity concentration (CC_50_) is the concentration required for being toxic to the cells at 50%

**Table 4:** The summary of the anti-parasitic effects of the crude extracts of the *Q. infectoria* galls on in vitro and in vivo studies

Article	Extract	Parasite strain	Type of study	Methodology	Results	Outcome
Sawangjaroen et al. (37)	Methanol	*Entamoeba histolytica* (2.0 × 10^4^ – 2.5 × 10^4^ troph/mL)	In vivostudy using female Swiss albino mice, weighing 25–35 g, aged 1–1.5 months (*n* = 110)	Mice were randomly grouped into: *Q. infectoria* - 125 mg/kg (*n* = 15) *Q. infectoria* - 250 mg/kg (*n* = 15) *Q. infectoria* - 500 mg/kg (*n* = 15) *Q. infectoria* - 1000 mg/kg (*n* = 15) Metronidazole - 62.5 mg/kg (*n* = 15) Metronidazole - 125 mg/kg (*n* = 15) Untreated control (*n* = 15) – Mice were inoculated with *E. histolytica* by an intraperitoneal injection prior to treatment. – Treatments were given daily p.o for five consecutive days. – Caecal samples were dissected at the end of the treatment for macroscopic and microscopic examination. Parameters: Average caecal score (contents, walls) Number of mice cured	Scores of caecal content and caecal wall exposed to different doses of the extract were higher than the metronidazole control group The extract significantly decreased infection with curation compared to metronidazole	The extract appeared to be effective against caecal amoebiasis in mice
Sawangjaroen and Sawangjaroen (23)	Methanol	*Blastocystis hominis* (10^5^ cells/mL)	In vitro	Test preparation as follows: *Q. infectoria* (62.5–2000 μg/mL) Metronidazole, a standard control (1.25–40 μg/mL) DMSO, a positive control – Test tubes containing egg slant and tested sample (*Q. infectoria* or control drug) at different concentrations were mixed with *B. hominis* and incubated for 48 h at 37 °C – The result was reported as inhibited, moderately inhibited or not inhibited – Confirmation for parasite killing and growth inhibition occurred in a new fresh medium at another 48 h incubation Parameters: Killing concentration at 50% (KC_50_) Effective concentration to inhibit growth at 50% (EC_50_)	2000 μg/mL of the extract was able to kill 67% and inhibited 76% of the parasite growth KC_50_ = 1,248 μg/mL EC_50_ = 171 μg/mL	The extract is evident in reducing infection against *B. hominis*
Ozbilgin et al. (35)	n-hexane Methanol	*Blastocytis* spp. isolates (10^5^ cells/mL)	In vitro	Test preparation as follows: *Q. infectoria* (62.5–4000 μg/mL) Metronidazole, a standard control (0.6–40 μg/mL) Saline solution, as a positive control – Test tubes containing saline solution and test sample (*Q. infectoria* or control drug) were added with *Blastocystis* spp and cultivated for 48 h at 37 °C – Each tube was checked for the presence of living cells Parameter: Effective concentration to inhibit growth at 50% (EC_50_)	n-hexane EC_50_ = 3.45 × 10^6^ μg/mL (inactive activity) Methanol EC_50_ = ~ 336.8 μg/mL	Both extracts reduce blastocystis but significantly lower with the control drug (*P* < 0.05)
Kheirandish et al. (36)	Methanol	*Leishmania major* (2 × 10^6^ cell/mL)	In vitro	Test preparation against promastigotes as follows: *Q. infectoria* (0–80 μg/mL) Glucantim (MA), a standard control (0–125 μg/mL) Positive control – Promastigotes were treated with test samples except for blank and incubated for 72 h at 25 1 °C – The treated samples were added with MTT (according to MTT assay protocol) and measured at 570 nm to determine the antipromastigote activity Parameter: Inhibitory concentration to inhibit growth at 50% (IC_50_) Test preparation against amastigotes as follows: *Q. infectoria* (0–80 μg/mL) MA (0–125 μg/mL) Positive control Negative control – Prior to treatment, adherent macrophages (5 × 10^4^ cell/well) were prepared and infected with promastigotes for 4 h (37 °C, 5% CO_2_) – The treatments then proceeded for another 24 h and incubated for 24 h, 48 h and 72 h – The assessment was done using Giemsa-fixed methanol smear Parameter: Inhibitory concentration to inhibit growth at 50% (IC_50_) – Assessment of promastigote invasion into macrophages was done – Promastigotes were pre-incubated with *Q. infectoria* (2.5 μg/mL) for 2 h and re-incubated with macrophages for another 4 h – The tested samples were evaluated using Giemsa-stained smears to assess the occurrence of infection Parameter: Percentage of infected macrophage (%)	For the anti-promastigote activity, the IC_50_ value of the extract was 12.65 μg/mL, while IC_50_ value for anti-amastigote activity was 10.31 μg/mL Promastigotes upon treatment with *Q. infectoria* were able to infect 33.2% of the macrophages compared to control without drug (76.5%) All parameters recorded showed that treatments with *Q. infectoria* were effective in reducing infection caused by *L. major* compared with the untreated control containing parasite (*P* < 0.05)	The extract was able to reduce cutaneous leishmaniasis in vitro
Kheirandish et al. (36)	Methanol	*Leishmania major* (2 × 10^6^ cell/mL)	In vivo Male BALB/c mice aged 6–8 weeks old (*n* = 84)	Mice were randomly grouped into: Non-infected and non-treated (*n* = 12) Infected, but non-treated (control group) (*n* = 12) Non-infected and treated with 20 mg/kg *Q. infectoria* (*n* = 12) Infected and treated with 20 mg/kg *Q. infectoria* (*n* = 12) Infected and treated with 10 mg/kg *Q. infectoria* (*n* = 12) Infected and treated with 5 mg/kg *Q. infectoria* (*n* = 12) Infected and treated with 60 mg/kg/day meglumine antimoniate, glucantime (MA) (*n* = 12) – Mice were inoculated subcutaneously with L. major (stationary phase promastigote) at the base of the tails prior to treatment – Treatments were given for 6 weeks and development of lesion at the site of parasite inoculation were assessed Parameters: Measurement of lesion size Microscopic examination of lesions Assessment of parasite burden	The number of parasites and parasite load of pooled draining lymph nodes decreased significantly upon treatment with *Q. infectoria* (*P* < 0.05) Mean diameter of the lesions was reduced according to the concentration-dependent manner after treatment of *Q. infectoria* at 5 mg/kg, 10 mg/kg and 20 mg/kg, respectively	The extract has a potential to replace MA in suppressing leishmanial infection
